# An Unexpected Diversity of Photoreceptor Classes in the Longfin Squid, *Doryteuthis pealeii*

**DOI:** 10.1371/journal.pone.0135381

**Published:** 2015-09-09

**Authors:** Alexandra C. N. Kingston, Trevor J. Wardill, Roger T. Hanlon, Thomas W. Cronin

**Affiliations:** 1 Department of Biological Sciences, University of Maryland Baltimore County, 1000 Hilltop Circle, Baltimore, Maryland 21250, United States of America; 2 Department of Physiology, Development, and Neuroscience, University of Cambridge, Cambridge, CB2 3EG, United Kingdom; 3 Marine Biological Laboratory, Woods Hole, Massachusetts, 02543, United States of America; University Zürich, SWITZERLAND

## Abstract

Cephalopods are famous for their ability to change color and pattern rapidly for signaling and camouflage. They have keen eyes and remarkable vision, made possible by photoreceptors in their retinas. External to the eyes, photoreceptors also exist in parolfactory vesicles and some light organs, where they function using a rhodopsin protein that is identical to that expressed in the retina. Furthermore, dermal chromatophore organs contain rhodopsin and other components of phototransduction (including retinochrome, a photoisomerase first found in the retina), suggesting that they are photoreceptive. In this study, we used a modified whole-mount immunohistochemical technique to explore rhodopsin and retinochrome expression in a number of tissues and organs in the longfin squid, *Doryteuthis pealeii*. We found that fin central muscles, hair cells (epithelial primary sensory neurons), arm axial ganglia, and sucker peduncle nerves all express rhodopsin and retinochrome proteins. Our findings indicate that these animals possess an unexpected diversity of extraocular photoreceptors and suggest that extraocular photoreception using visual opsins and visual phototransduction machinery is far more widespread throughout cephalopod tissues than previously recognized.

## Introduction

Cephalopods are well known for their remarkable ability to transform their appearance by altering their dermal coloration, patterning, and shape. They are thought to achieve this by detecting visual scenes using their eyes and controlling their dynamic patterning in a top-down manner. In this model, eyes detect scenes and the brain initiates signals that result in dermal color and/or shape change [1,2] using chromatophores, iridophores, leucophores, and papillae [3,4]. It has also been shown that several cephalopod species have extraocular photoreceptors in parolfactory vesicles and epistellar bodies, both in the central nervous system[5]. These are functional partly because of the translucent skin of cephalopods. Some cephalopods also have photoreceptors outside the CNS [6]. For example, squid and cuttlefish have visual opsins in their skin that are thought to contribute to light detection and possibly to dermal patterning [7].

Extraocular, or non-visual, photoreceptors are light detectors that exist outside of eyes and do not contribute to image analysis. In cephalopods, all extraocular photoreceptors that have been described are thought to detect light using rhodopsin, identical to the form expressed in the eye. Some are also known to use other visual phototransduction components, including retinochrome, Gqα, visual arrestin, and rhodopsin kinase [6, 7,8]. The photopigments rhodopsin and retinochrome are of special interest. Rhodopsin is formed when an opsin protein is covalently bound to a vitamin-A derived chromophore. When the chromophore accepts a photon, a signaling cascade is initiated that results in the opening or closing of ion channels, and ultimately, a neural signal (reviewed in [9]). Retinochrome, a photoisomerase expressed in cephalopod retinas, is thought to convert the all-*trans* chromophore to the 11-*cis* configuration to regenerate active rhodopsin [1,2,9].

The deep sea squid, *Todarodes pacificus*, has photoreceptors located in small parolfactory vesicles along the optic tract [3,4,10]. While the physiological function of these photoreceptive cells is not well understood, they are known to respond electrically to light and to express rhodopsin and retinochrome, two proteins essential to vision in the retina [5,10,11]. The bobtail squid, *Euprymna scolopes*, has extraocular photoreceptors located in its light organ, thought to be involved in a feedback mechanism to regulate the luminance levels emitted from the organ [12]. These light organ photoreceptors express rhodopsin, arrestin, and rhodopsin kinase proteins and respond electrically to light stimuli [6]. The epistellar body of the curled octopus, *Eledone cirrhosa*, has photoreceptor cells that respond to light electrophysiologically with a spectral sensitivity similar to that of rhodopsin [7,11]. Additionally, molecular studies of putative distributed photoreceptors in the skin of *Sepia officinalis*, *Sepia latimanus*, and *Doryteuthis pealeii* have shown that transcripts of several phototransduction machinery genes, including rhodopsin, retinochrome, Gqα, and squid TRP channel, are expressed throughout dermal tissues. Significantly, rhodopsin, retinochrome, and Gqα proteins are all expressed in chromatophore membranes, radial muscle fibers, and sheath cells, all of which are components of chromatophore organs that are used in signaling and camouflage [6,7,10]. Wherever they have been characterized in extraocular photoreceptors of cephalopods, rhodopsin transcripts are identical to those located in retinas of the same species.

In this study, we investigated rhodopsin and retinochrome antibody labeling throughout the body of adult and hatchling longfin squid, *Doryteuthis pealeii*, using a modified whole-mount immunohistochemical technique and confocal imaging. We confirm that these antibodies correctly label the retina, dermal chromatophores, and paralarval parolfactory vesicles and identify four new tissue regions where rhodopsin and retinochrome antibodies label. We suggest that these regions contain novel, previously undescribed photoreceptors that operate using rhodopsin and retinochrome proteins. While the functionality of these four classes of putative photoreceptors is currently unknown, we will discuss hypotheses and propose possible functions.

## Materials and Methods

### Tissue collection and fixation

*Doryteuthis pealeii* adults were collected by brief slow trawling runs in Vineyard Sound (41°N 26’ 30”N, 070° 46’ 28”W) by the Aquatic Resources Division at Marine Biological Laboratory (MBL) in Woods Hole, MA USA. Fertilized egg masses were collected from squid holding tank populations and isolated in mesh bottom enclosures that were floating in running sea water tanks. Two to four days post-hatching, hatchlings were collected. Hatchlings, also noted in the literature as paralarvae, are young developing cephalopods that have recently hatched from their egg casings [13]. From large holding tank populations, individuals that showed no evidence of skin damage in at least one fin were transferred to separate holding tanks for males and females, respectively. Each squid was fed daily with small live fish (*Fundulus* spp.), and animals were kept for up to two weeks before being used for experiments.

Prior to fixation, adult *D*. *pealeii* were anaesthetized by immersion in seawater containing a sublethal concentration of ethanol (3%), and then killed by decapitation and decerebration (following institutional guidelines). Live hatchlings in seawater were isolated into a falcon tube and fixative added directly. All tissues were prepared for immunohistochemistry by fixation in 4% paraformaldehyde in PBS (0.1 M phosphate buffered saline, pH 8). Following dissection, adult tissue was fixed for four hours at room temperature. Hatchlings were fixed whole for one hour at room temperature. After fixation, tissues were stored in PBS at 4°C until use.

Adult tissues used for whole-mount immunohistochemistry include retina, dorsal mantle, and fin. After fixation, adult retinas were vibratome sectioned at approximately 150 μm. Adult dorsal mantle sections were cut with a straightedge razor blade into approximately 5 mm^2^ pieces, and all layers of dermal tissue were separated from underlying mantle muscle tissue after fixation. Intact adult fin tissue was cut with a vibratome at approximately 600 μm from a whole fin to expose all layers from ventral to dorsal epidermis. Hatchlings were labeled and imaged intact.

### Antibodies

Antibodies used in this study included custom-made anti-cephalopod rhodopsin and anti-cephalopod retinochrome, and commercially available anti-acetylated alpha-tubulin (Sigma). Western blots and previous immunohistochemical labeling in thin sections, including extensive controls, show that these antibodies label the proteins against which they were designed [7]. The antibody directed against the first fifteen predicted amino acids of rhodopsin transcripts from *S*. *officinalis*, *S*. *latimanus*, and *D*. *pealeii* will hereafter be referred to as “rhodopsin antibody”. The antibody directed against the predicted terminal eleven amino acids of retinochrome transcripts from *S*. *officinalis*, *S*. *latimanus*, and *D*. *pealeii* will hereafter be referred to as “retinochrome antibody”. Anti-acetylated alpha tubulin specifically labels nerves in cephalopods [14].

Secondary antibodies included AlexaFluor 555 goat anti-chicken, AlexaFluor 594 goat anti-mouse, and AlexaFluor 633 goat anti-rabbit (LifeTechnologies). These secondary antibodies were chosen because short-wavelength autofluorescence is commonly observed when imaging thick sections of cephalopod tissue, and these secondary antibody excitation and emission wavelengths avoid this phenomenon [15].

### Immunolabeling

Immunolabeling was performed by modifying a whole-mount immunolabeling protocol from Gonzalez-Bellido and Wardill [15]. All tissues were dehydrated in an ethanol series (ethanol in PBS) of 30, 50, 70, 90, and 100% for twenty minutes each, at room temperature, to remove lipids. Tissues were rehydrated in 90, 70, 50, and 30% ethanol series for twenty minutes each, at room temperature. Tissues were washed in PBS three times for ten minutes at room temperature, and blocked in PBS-TX (0.1M PBS+0.3% Triton-100; Sigma) plus 10% normal goat serum (NGS; Vector Labs) for two hours at room temperature. Primary antibodies were diluted at a concentration of 1:100 in 1ml PBS-TX+10% NGS and added to tissues contained in a 24-well plate. Primary antibody incubations lasted four days at 4°C. Tissues were washed in PBS-TX+10% NGS for one hour, three times, at room temperature. Secondary antibodies were diluted at a concentration of 1:50 in 1ml PBS-TX+10% NGS and applied to tissues. Secondary antibody incubations lasted three days at 4°C. Tissues were washed in PBS three times for thirty minutes at room temperature in the dark. Tissues were cleared in a thiodiethanol (TDE; 2’2’-thiodiethanol in PBS; Sigma) series of 10, 20, 30, 40, 50, 60, 70, 80, 90, and 97% for one hour each, at room temperature, in the dark, and mounted in 97% TDE on 600μm-thick stainless steel slides with a cover slip on each side of a circular punch through the middle of the slide. Cover slips were sealed to the slide with nail polish.

### Confocal microscopy, tissue visualization, and image processing

A Zeiss LSM 780 was used to image tissues. Images were taken using a 25x long-working-distance multi-immersion objective, set to “oil”. Retinal tissue was visualized in longitudinal section to expose the length of the photoreceptor cells and to visualize the inner and outer segments of photoreceptors of the retina, as well as the nervous tissue at the back of the eye. Dorsal mantle tissue was visualized from the epidermal layer through the chromatophore and iridophore layers, to underlying muscle tissue, i.e. *en face*. Fin tissue was visualized in cross-section, where the fin was cut from ventral to dorsal surfaces to include the ventral and dorsal dermal tissues and the muscle tissue that connects the dermal tissues. Hatchlings were imaged from the ventral surface inward.

In our images, rhodopsin immunolabeling appears green, retinochrome labeling appears red, and areas where both antibodies colabel the same tissue appear yellow. Anti-acetylated α-tubulin antibody appears white. In all images, autofluorescence of the tissue was imaged using a 405 nm laser and appears blue. Visualization of autofluorescence allows imaging of structural components of cephalopod tissue [15]. All secondary-only control images are shown in Supporting Data.

Images were processed using Fiji (Image J). Maximum-intensity images were created from z-stacks (Z-projection). Access to all the images and raw data for this paper can be found at http://dx.doi.org/10.1575/1912/7383 in the Woods Hole Open Access Server (WHOAS). This material consists of images and data that support each of figures in this paper and the supplement.

### Ethics Statement

In the USA, squids are not covered under IACUC guidelines however we continue to match international ethical guidelines in the treatment and handling of all animals used within this study. Squids were collected by trawling by the Aquatic Resources Division at Marine Biological Laboratory in Woods Hole, MA. The authors were not directly involved in collection of adult squids. Adults were housed in running sea water tanks until use, and were sacrificed by decapitation and decerebration immediately upon collection from tanks. The authors collected paralarvae from running sea water tanks in which they hatched, and they were immediately fixed alive. Traditional anesthetization of cephalopods using ethanol was not appropriate for paralarvae, as immersion in ethanol causes juveniles to be disoriented and swim into the walls of their container, damaging dermal tissues and sensory hair epithelia. The MBL has a collection permit for *Doryteuthis pealeii* from the Massachusetts Division of Marine Fisheries, which is renewed annually. This species is not endangered in MA and is still collected commercially.

## Results

### Retina

**Fig 1:**
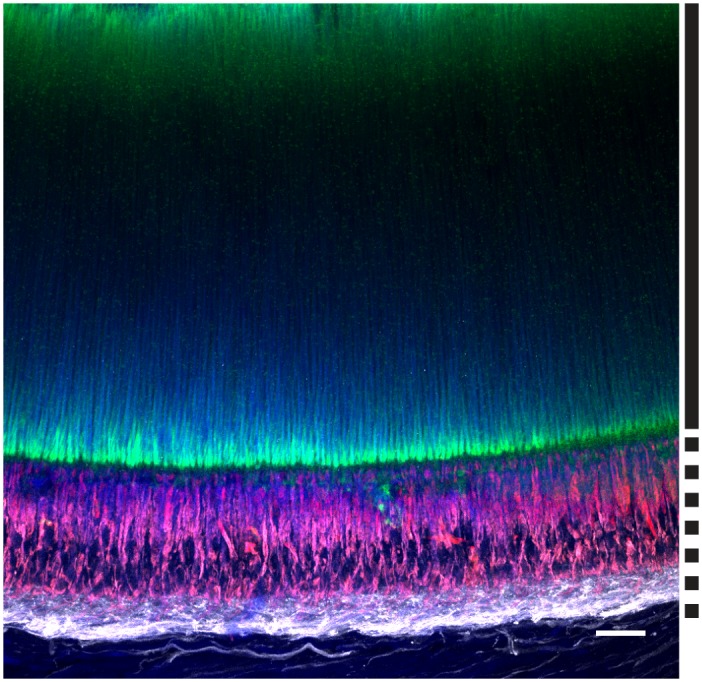
Immunolabeling of rhodopsin, retinochrome, and acetylated α-tubulin in a whole mount of *D*. *pealeii* retina. (A) Rhodopsin (green) is present only in outer segments, represented by regions parallel to the solid line. Rhodopsin is seen predominantly on the tips and bases of outer segments due to dense packing of visual and screening pigments, which prevent binding in parts of the outer segments. (B) Retinochrome (red) is present only in the inner segments, indicated by the dashed line. (C) Acetylated α-tubulin (white) is present in the inner segments. Blue shows autofluorescence of the tissue. Scale bar, 25μm.

Rhodopsin antibody labeled outer segments of the photoreceptor cells in the retina of adult *D*. *pealeii* (Fig 1). Retinochrome antibody labeled the inner segments of photoreceptor cells. Acetylated α-tubulin antibody labeled the proximal portion of the retina, where axons leave photoreceptor cells. Blue labeling seen in the outer segments represents autofluorescence of that tissue. Areas where rhodopsin labeling in the outer segments appears blue-green are the result of rhodopsin labeling and autofluorescence in the same region of outer segments. These results serve as a positive control for the protocol and provide confirmation that antibodies label the proteins of interest, as expression of rhodopsin and retinochrome in cephalopod retinas are well known [7,16], and that these antibodies label with the same pattern as thinly cryo-sectioned *D*. *pealeii* retinas [7,8]. α-tubulin labeling of axons at the proximal portion of the retina also serves as a positive control, confirming that the antibody labels nerves. Secondary-only control shows a lack of non-specific labeling in the retina (S1 Fig).

### Dorsal mantle

**Fig 2:**
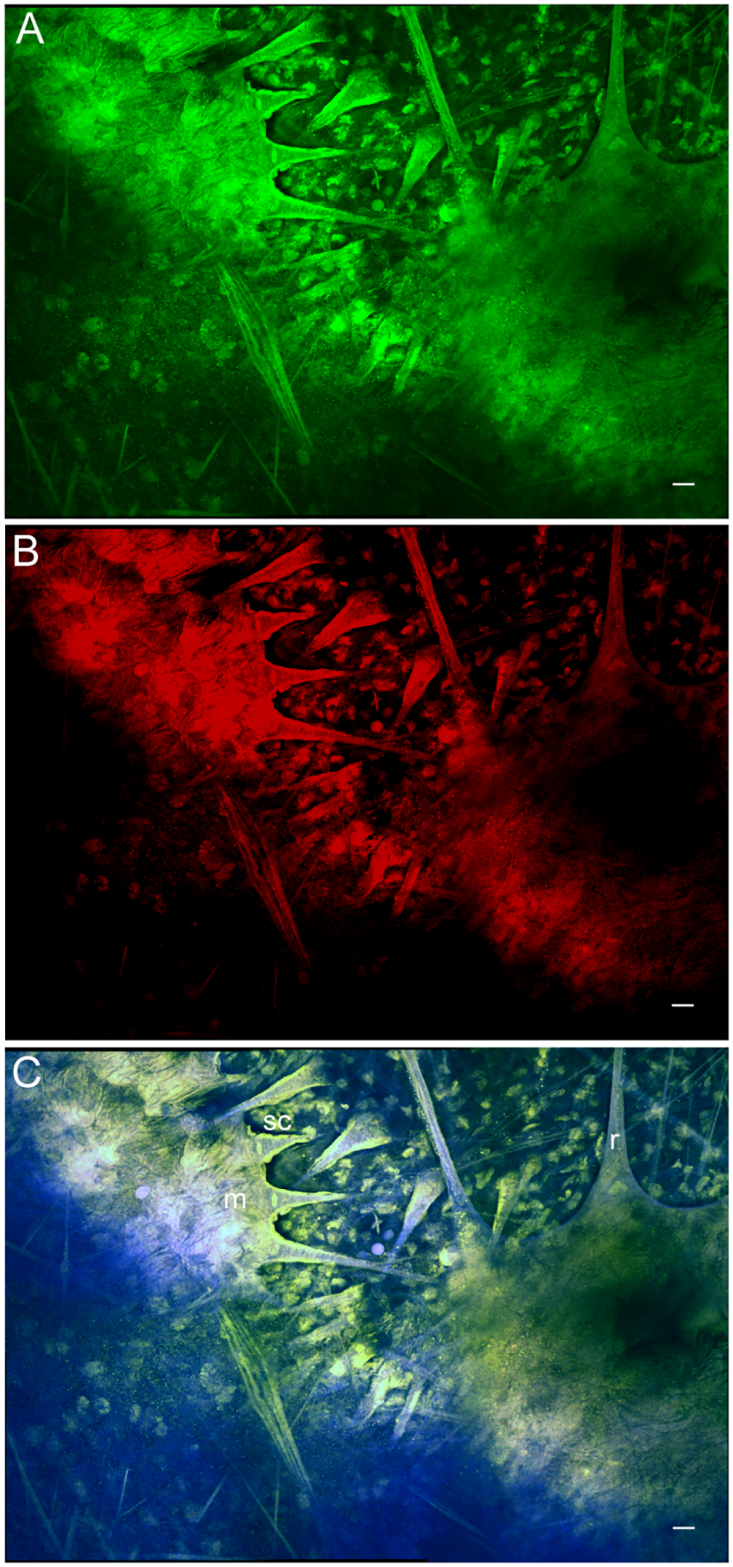
*D*. *pealeii* dorsal mantle tissue immunolabeled with anti-rhodopsin and anti-retinochrome, and imaged in the chromatophore layer. (A) Rhodopsin (green) labeling only; (B) retinochrome (red) labeling only. (C) Overlap of rhodopsin and retinochrome labeling in the chromatophore membrane (m), radial muscle fibers (r), and sheath cells (sc) appears yellow. Blue shows autofluorescence of the tissue. Scale bar, 25μm.

Adult *D*. *pealeii* dorsal mantle tissue showed labeling with antibodies directed against rhodopsin and retinochrome in several distinct components of the chromatophore organs (Fig 2). These components included the pigment cell membrane that surrounds the pigment sac, the radial muscle fibers that attach directly to this sac, and the sheath cells that surround the radial muscles and sac. These results complement and are consistent with our earlier studies of dorsal mantle tissue in thin cryosection, where rhodopsin and retinochrome antibodies also labeled chromatophore pigment sacs, radial muscle fibers, and sheath cells [7]. Secondary-only control shows a lack of non-specific labeling in mantle tissue (S3 Fig).

### Fin

**Fig 3:**
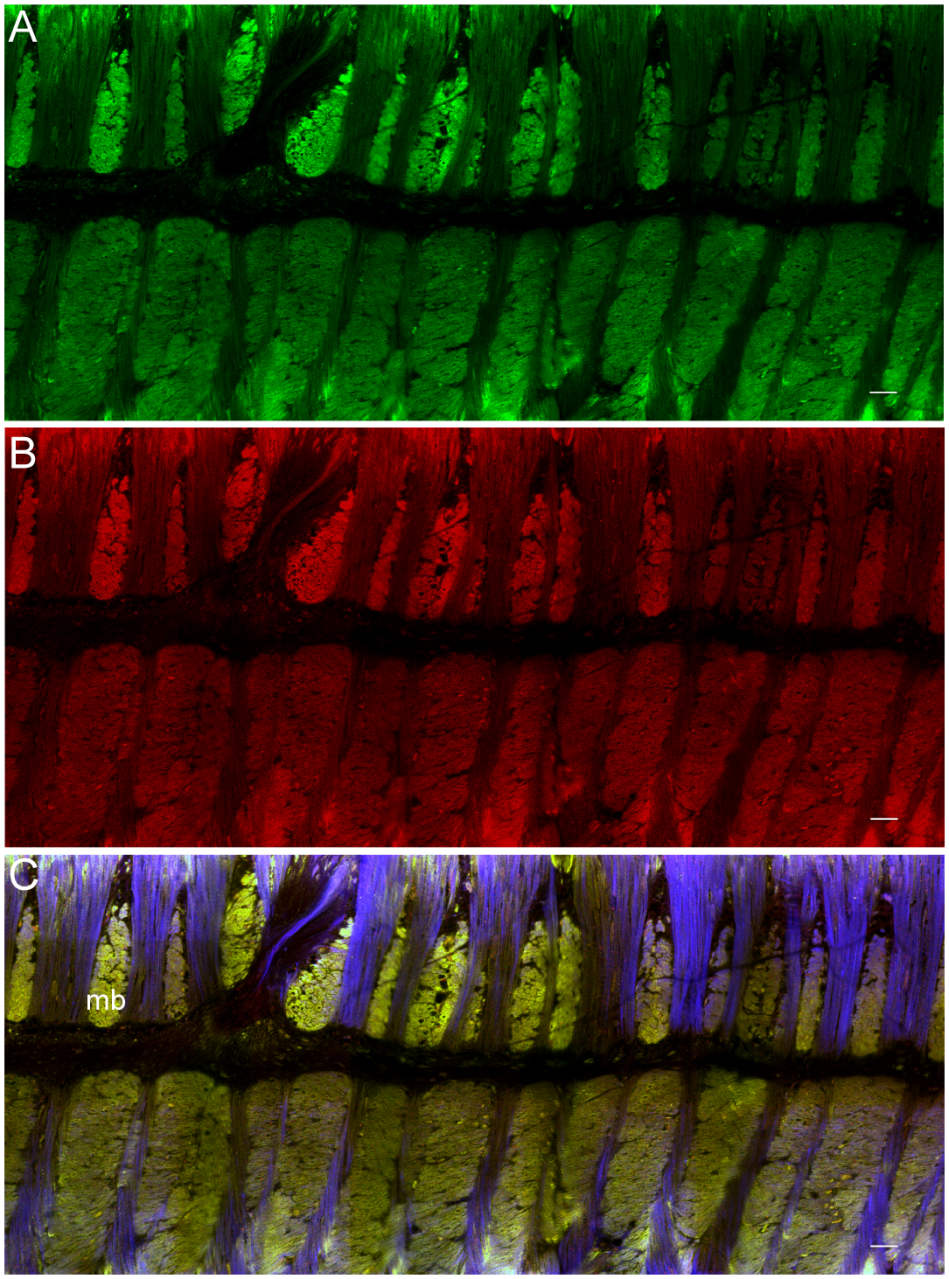
Immunolabeling of rhodopsin and retinochrome in a *D*. *pealeii* central fin muscle cross-section. (A) Rhodopsin (green) labeling only; (B) retinochrome (red) labeling only. (C) Overlap of rhodopsin and retinochrome labeling in large muscle bundles (mb) parallel to dermal tissue appears yellow. Blue shows autofluorescence of the tissue. Scale bar, 25μm.

Adult fin tissue showed colabeling of rhodopsin and retinochrome in one set of central muscle bundles (Fig 3). The central muscle bundles are formed from large muscle bundles that span the entire width of the fin and are separated at the midline [17]. Muscle bundles run both parallel and perpendicular to the dermal surfaces of the fin (as seen in Fig 3), but only bundles running parallel to the dermal surface showed colabeling. In contrast, vertical muscles, perpendicular to the dermal surface of the fin, remained free of either label. The secondary-only control shows a lack of non-specific labeling in fin muscle (S3 Fig).

### Hatchling parolfactory vesicles

**Fig 4:**
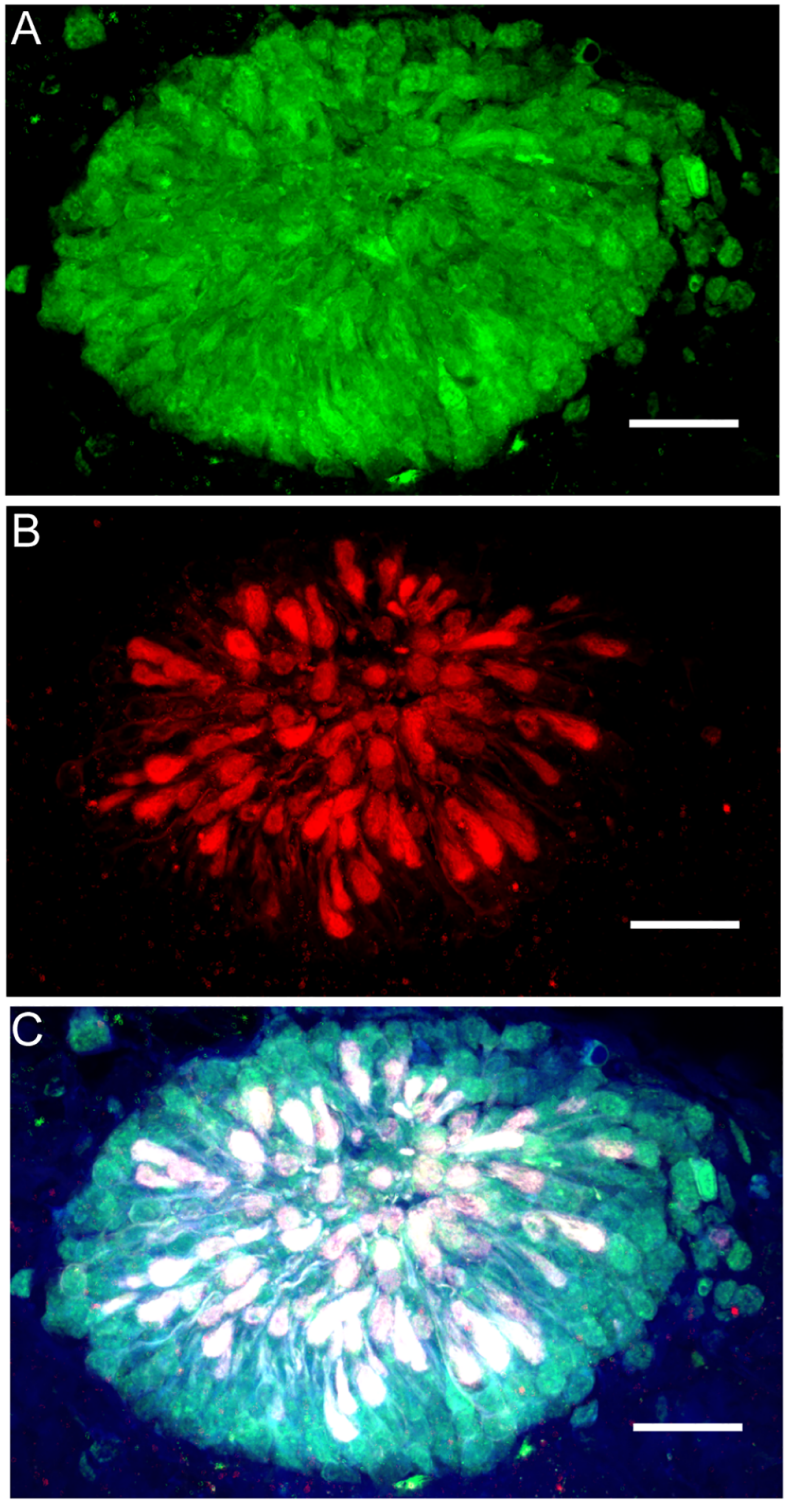
*D*. *pealeii* paralarval parolfactory vesicle immunolabeled with anti-rhodopsin, anti-retinochrome, and anti-acetylated α-tubulin antibodies. (A) Rhodopsin (green) is present throughout the parolfactory vesicles while (B) retinochrome (red) and α-tubulin (white) are present in discrete cell bodies. (C) Overlap of rhodopsin, retinochrome, and α-tubulin labeling appears white. Blue shows autofluorescence of the tissue. Scale bar, 25μm.

In *D*. *pealeii* hatchlings, portions of the parolfactory vesicles (structures lying ventrally to the optic tracts) labeled with rhodopsin, retinochrome, and acetylated α-tubulin antibodies (Fig 4). The entire parolfactory vesicle labeled positively for rhodopsin, while retinochrome and acetylated α-tubulin labeled cell bodies within the structure. The secondary-only control shows a lack of non-specific labeling in a hatchling parolfactory vesicle (S4 Fig).

### Hatchling arm ganglia and suckers

**Fig 5:**
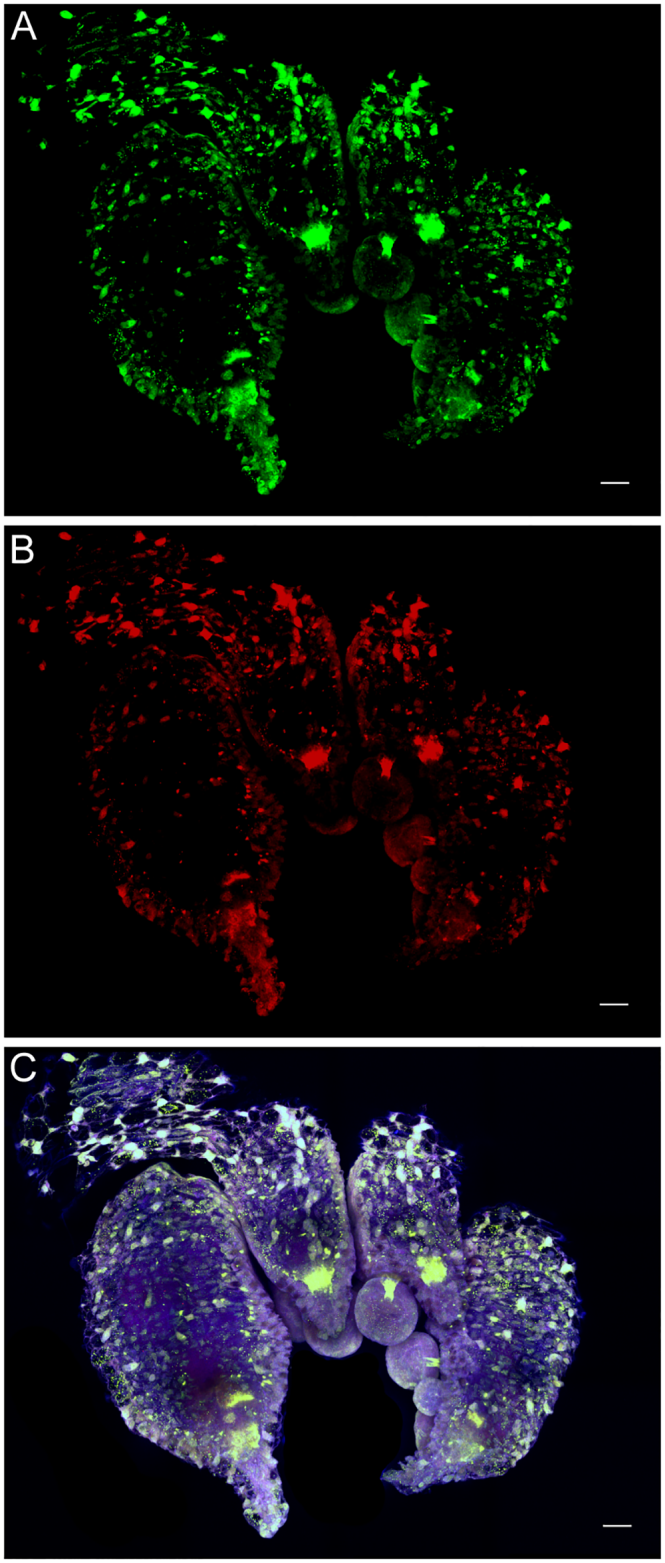
Immunolabeling in *D*. *pealeii* paralarval arms shows presence of rhodopsin and retinochrome in each arm ganglion. (A) Rhodopsin (green) labeling only; (B) retinochrome (red) labeling only. (C) Overlap of rhodopsin and retinochrome labeling occurs at the junction between arm and each sucker, and appears yellow. Small cell bodies also labeled with rhodopsin and retinochrome are hair cells (see Fig 6). Blue shows autofluorescence of the tissue. Scale bar, 25μm.

Two types of structures in each arm of *D*. *pealeii* colabeled with rhodopsin and retinochrome-specific antibodies (Fig 5). First, a single large ganglion near the base of each arm colabeled. In addition, numerous suckers join to the main structure of each arm by a ring of muscular tissue called the peduncle. Within each of the peduncles, both antibodies labeled two parallel thread-like structures, thought to be the peduncle nerves that extend from each sucker to nerves in the arm [18]. The secondary-only control shows a lack of non-specific labeling in hatchling arms and suckers (S5 Fig).

### Hair cells

**Fig 6:**
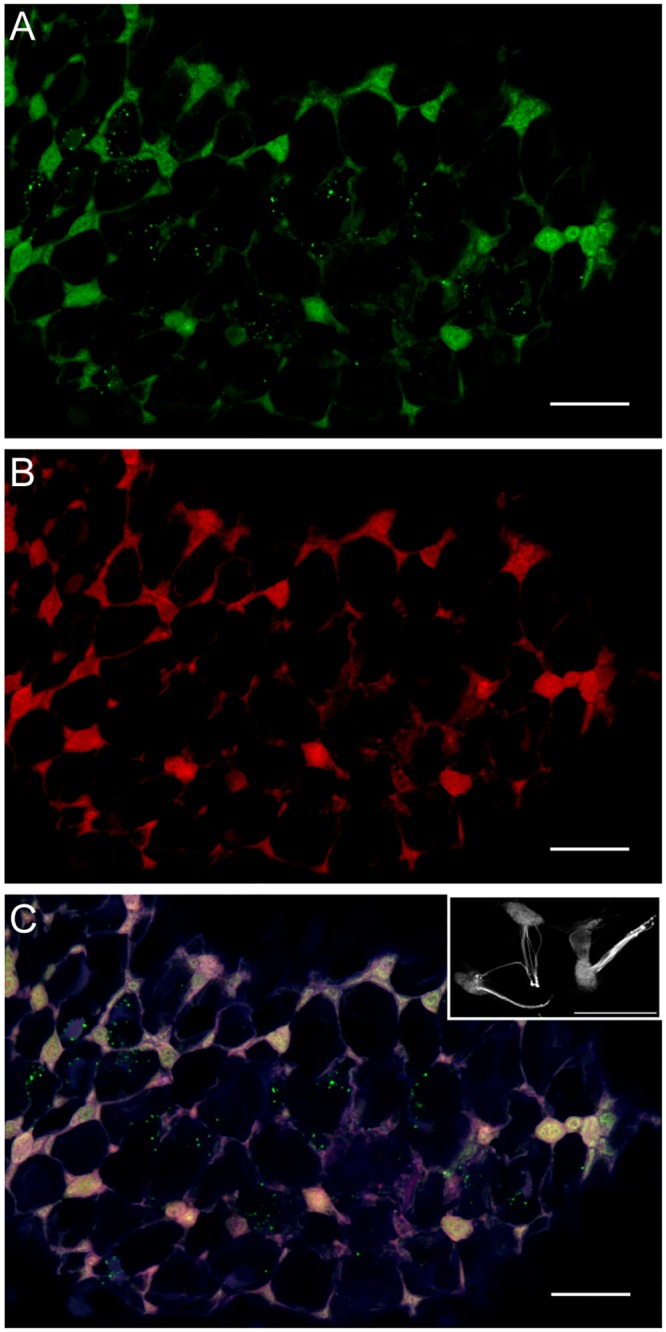
Hair cells in *D*. *pealeii* hatchlings immunolabeled with rhodopsin and retinochrome-specific antibodies. (A) Rhodopsin (green) labeling only; (B) retinochrome (red) labeling only. (C) Overlap of rhodopsin and retinochrome labeling in hair cell cell bodies appears yellow. Acetylated α-tubulin antibody (white) labels the cell body and cilia bundle protruding from the cell body (inset). Blue shows autofluorescence of the tissue. Scale bar, 25μm.

In hatchlings of *D*. *pealeii*, the entire dermal surface is covered with small hair cells. These hair cells possess cilia that project outward from the cell body [19]. The cells are thought to be mechanosensory; in fact, Mackie [13] called them “epidermal primary sensory neurons”. Hair cells of *D*. *pealeii* hatchlings contain several notable structures that appear to be photosensory. The cell bodies, which are spherical in shape, colabeled with antibodies directed against rhodopsin, retinochrome, and acetylated α-tubulin (Fig 6). In contrast, the ciliary tufts only labeled with acetylated α-tubulin antibody. The secondary-only control shows a lack of non-specific labeling in hatchling hair cells (S6 Fig).

## Discussion

We identified four types of putative photoreceptors in tissues that were not previously thought to have light-sensing abilities. In the retina, rhodopsin labeling is visualized at the tips and base of the outer segments, but little labeling is seen in the middle of the outer segments. This is due to dense packing of visual pigments and screening pigments, which prevents binding of antibodies [20]. Several components of chromatophore organs (e.g. pigment cell membranes, radial muscle fibers, and sheath cells) also labeled with antibodies against rhodopsin and retinochrome, a result that supports our earlier evidence for dermal photoreception in several cephalopod species [7]. Interestingly, the iridophores, which are neurally controlled structural reflectors [21,22] that lie subadjacent to the chromatophore layer [3,23], do not bind any of the antibodies we used. Iridophores, like chromatophores, contribute to dermal patterning in cephalopods [23,24]. It is therefore surprising that, in contrast to the chromatophores, iridophores are apparently devoid of light-detecting molecules. This apparent lack of photoreceptive molecules obviates their potential sensitivity to ambient light.

Kingston et al. [7] identified transcripts for several genes that are components of the phototransduction machinery in fin muscle tissue. We now find that only longitudinally arranged central muscle bundles of the fin label with rhodopsin and retinochrome-specific antibodies. It is not obvious why muscle tissue would be light sensitive. However, squid skin can become highly translucent with the contraction of chromatophores and a reduction in the reflectivity of iridophores [23,25]. At such times, light can penetrate into deeper structures, allowing photoreceptors in underlying tissues to be stimulated. The phototransduction machinery present in fin muscle is likely also to be expressed in mantle, arm, and head muscles. If so, muscle—or other—photoreceptors throughout the body may at times be active.

In the squid *Todarodes pacificus*, the parolfactory vesicles are small structures that lie behind the eye, near the optic tract, which express rhodopsin and retinochrome proteins simultaneously [10]. Similarly, parolfactory vesicles of *D*. *pealeii* hatchlings label for rhodopsin and retinochrome, as well as α-tubulin. While it has long been known that *D*. *pealeii* has light-sensitive parolfactory vesicles [26], this is the first published evidence that they contain rhodopsin and retinochrome. Oddly, we did not see labeling of rhabdomere-like structures within this tissue, as reported in *T*. *pacificus* [10]. Instead, cell bodies within the vesicles labeled with our antibodies. Hara and Hara [10] used electron microscopy to reveal finer structure in *T*. *pacificus* parolfactory vesicles than we see in *D*. *pealeii*. Also, *D*. *pealeii* parolfactory vesicles are smaller and less developed than those of *T*. *pacificus*, at least in hatchlings, which perhaps explains the labeling pattern we found.

We also identified a number of putative photoreceptors within the arms of *D*. *pealeii* hatchlings. Single ganglia near the base of each arm coexpress rhodopsin and retinochrome. Octopus have a single ganglion, called an axial ganglion, at a similar location in each arm that confers tactile information [27,28]. While axial ganglia have not been identified in adult squid, these paralarval structures could be similar to octopus axial ganglia. Each sucker peduncle has two thread-like structures that express rhodopsin and retinochrome, which appear to be the peduncle nerves that innervate sucker mechanoreceptors [18]. Our results suggest that these structures are also light sensitive. Perhaps these peduncle nerves are multimodal, fulfilling the dual roles of ambient light detection and relaying tactile information from suckers to the central nervous system. If so, the basal ganglia handle both light sensing and mechanosensory modalities.

Perhaps the most fascinating finding of this study is the discovery that a distributed array of interconnected hair cells in the outer epidermis is apparently photoreceptive, as the cell bodies of these primary sensory neurons [13] label with rhodopsin- and retinochrome-specific antibodies. Their axons form a nerve net in the dermis, with some axons extending to the stellate ganglia [13]. Interestingly, we see no evidence of rhodopsin or retinochrome labeling in these axons, in the stellate ganglia themselves, or in nerves extending to or from them. This leads us to suggest that epidermal primary sensory neurons are multimodal cells, with both photoreceptive and mechanoreceptive properties [13]. Multimodal sensory cells with the ability to detect pressure and irradiance, such as these, may be particularly useful when manipulating objects or concealing by means of camouflage.

## Conclusions

In summary, we have identified several novel types of what appear to be photoreceptors in numerous tissues of the longfin squid, *Doryteuthis pealeii*. The longitudinal bundles of central fin muscle, arm ganglia, sucker peduncle nerves, and epidermal hair cells all labeled with antibodies directed against rhodopsin and retinochrome. We also demonstrated that rhodopsin and retinochrome are present in *D*. *pealeii* parolfactory vesicles. These must be added to the chromatophore organs, which we recently showed to express components of phototransduction [7]. More research is necessary to show if, how, and why the abundant diversity of putative photoreceptors identified throughout the body of *D*. *pealeii* actually sense light.

## Supporting Information

S1 Fig Secondary-only control immunolabeling in a whole mount *D*. *pealeii* retina, labeled with anti-rabbit 488 and anti-chicken 633 to ensure no non-specific labeling of secondary antibodies. Blue represents autofluorescence excited by a 405nm laser. Scale bar, 25μm.
(TIF)

S2 Fig Secondary-only control immunolabeling of *D*. *pealeii* dorsal mantle tissue, labeled with anti-rabbit 488 and anti-chicken 555 to ensure no non-specific labeling of secondary antibodies. Blue represents autofluorescence excited by a 405nm laser. Scale bar, 25μm.
(TIF)

S3 Fig Secondary-only control immunolabeling of *D*. *pealeii* central fin muscle cross-section, labeled with anti-rabbit 488 and anti-chicken 555 to ensure no non-specific labeling of secondary antibodies. Blue represents autofluorescence excited by a 405nm laser. Scale bar, 25μm.
(TIF)

S4 Fig Secondary-only control immunolabeling in a *D*. *pealeii* hatchling parolfactory vesicle, labeled with anti-rabbit 488 and anti-chicken 555 to ensure no non-specific labeling of secondary antibodies. Blue represents autofluorescence excited by a 405nm laser. Scale bar, 25μm.
(TIF)

S5 Fig Secondary-only control immunolabeling in *D*. *pealeii* hatchling arms, labeled with anti-rabbit 488 and anti-chicken 555 to ensure no non-specific labeling of secondary antibodies. Blue represents autofluorescence excited by a 405nm laser. Scale bar, 25μm.
(TIF)

S6 Fig Secondary-only control immunolabeling of hatchling hair cells in *D*. *pealeii*, labeled with anti-rabbit 488 and anti-chicken 555 to ensure no non-specific labeling of secondary antibodies. Blue represents autofluorescence excited by a 405nm laser. Scale bar, 25μm.
(TIF)
