# Supplementary figures and images for: An Unexpected Diversity of Photoreceptor Classes in the Longfin Squid, Doryteuthis pealeii
Source: PLoS One. 2015 Sep 9;10(9):e0135381. doi: 10.1371/journal.pone.0135381 (PMC4564192; doi:10.1371/journal.pone.0135381)

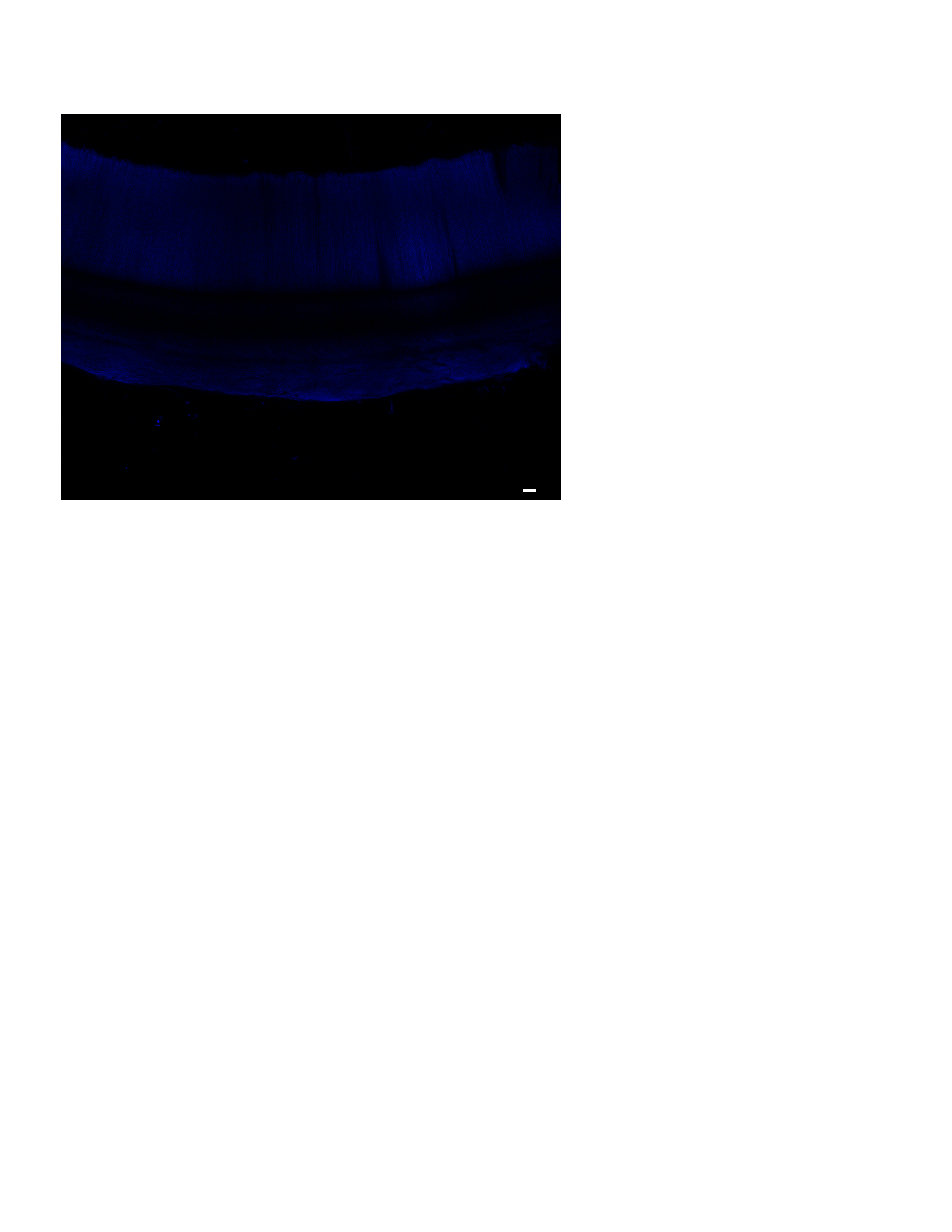

Supplement: S1 Fig — Blue represents autofluorescence excited by a 405nm laser. Scale bar, 25μm. (TIF) [file pone.0135381.s001.tif]

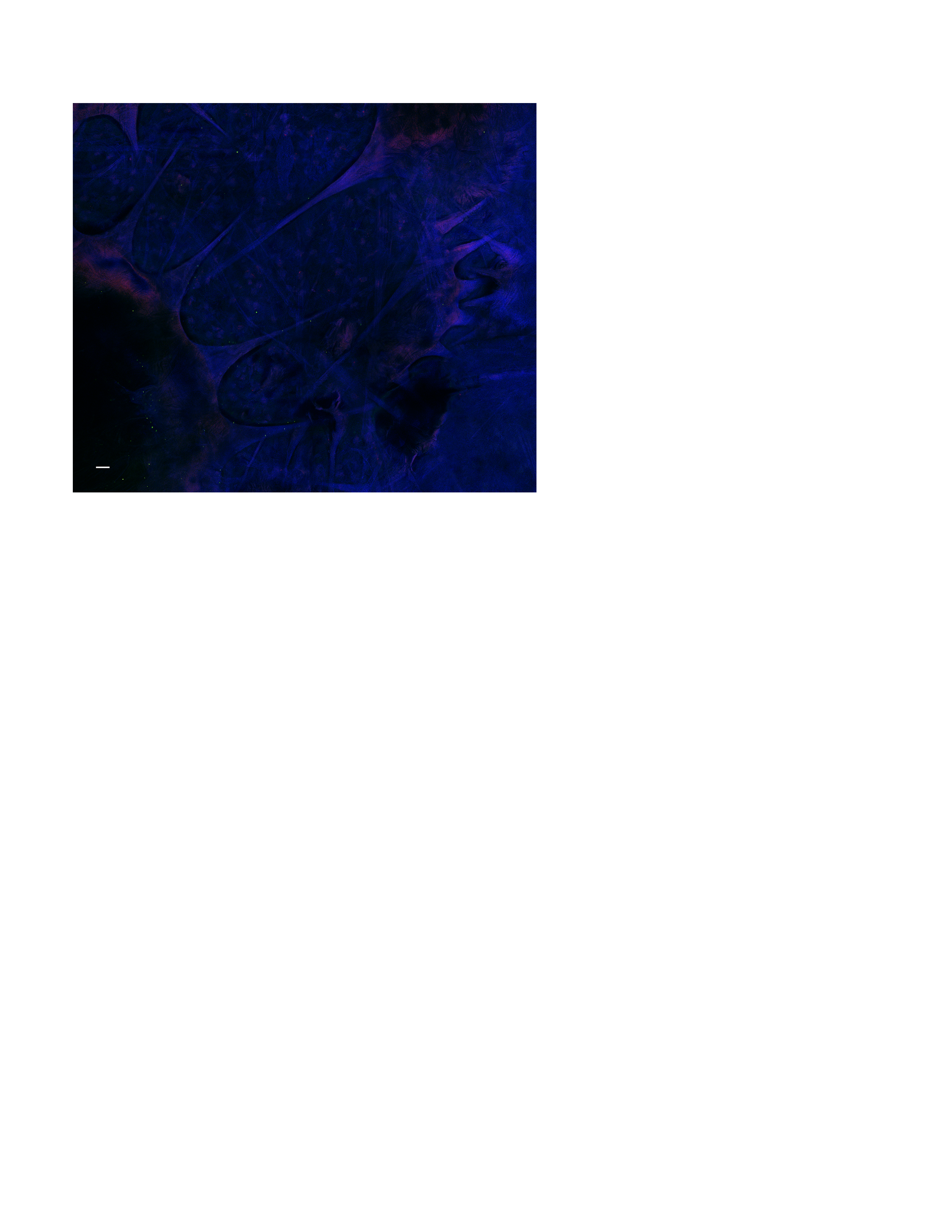

Supplement: S2 Fig — Blue represents autofluorescence excited by a 405nm laser. Scale bar, 25μm. (TIF) [file pone.0135381.s002.tif]

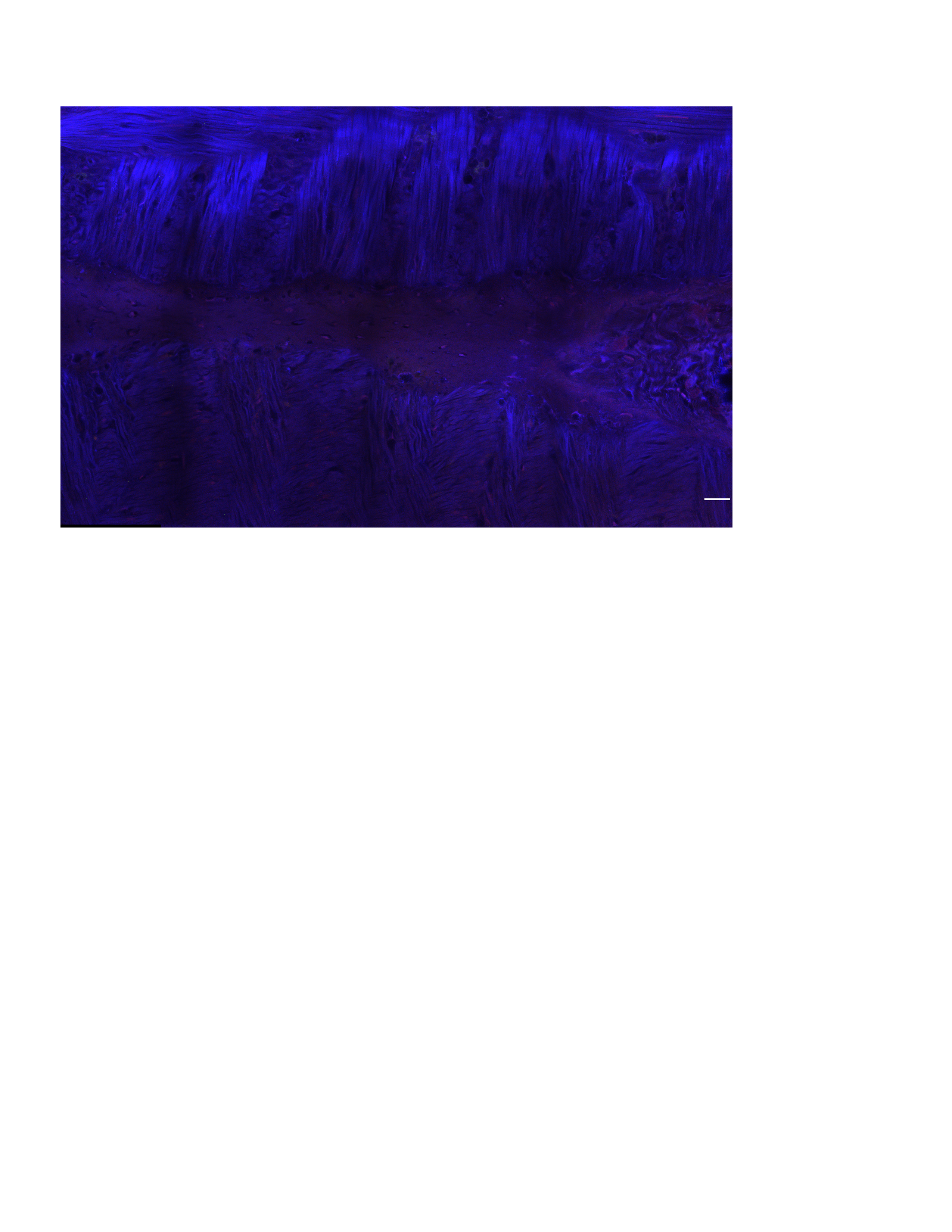

Supplement: S3 Fig — Blue represents autofluorescence excited by a 405nm laser. Scale bar, 25μm. (TIF) [file pone.0135381.s003.tif]

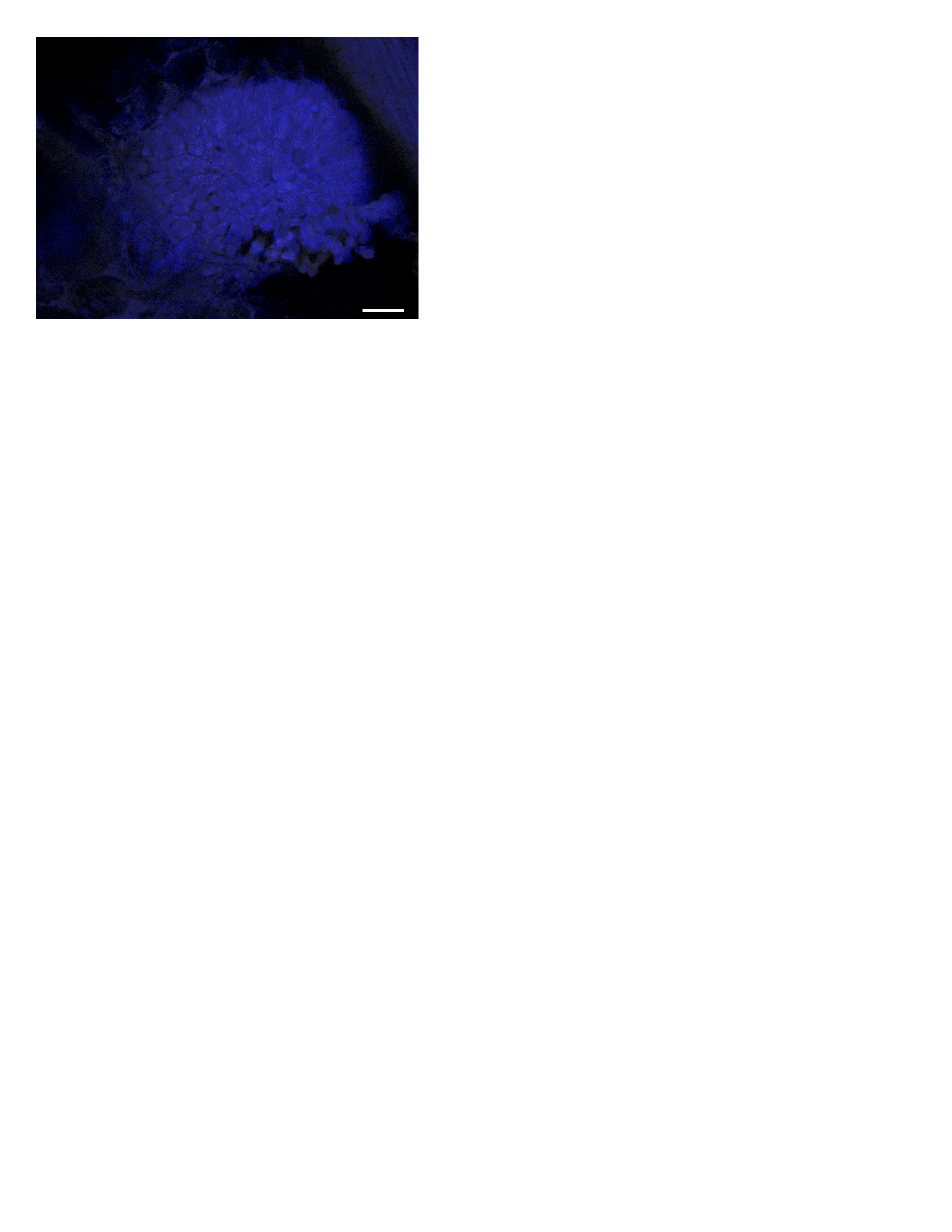

Supplement: S4 Fig — Blue represents autofluorescence excited by a 405nm laser. Scale bar, 25μm. (TIF) [file pone.0135381.s004.tif]

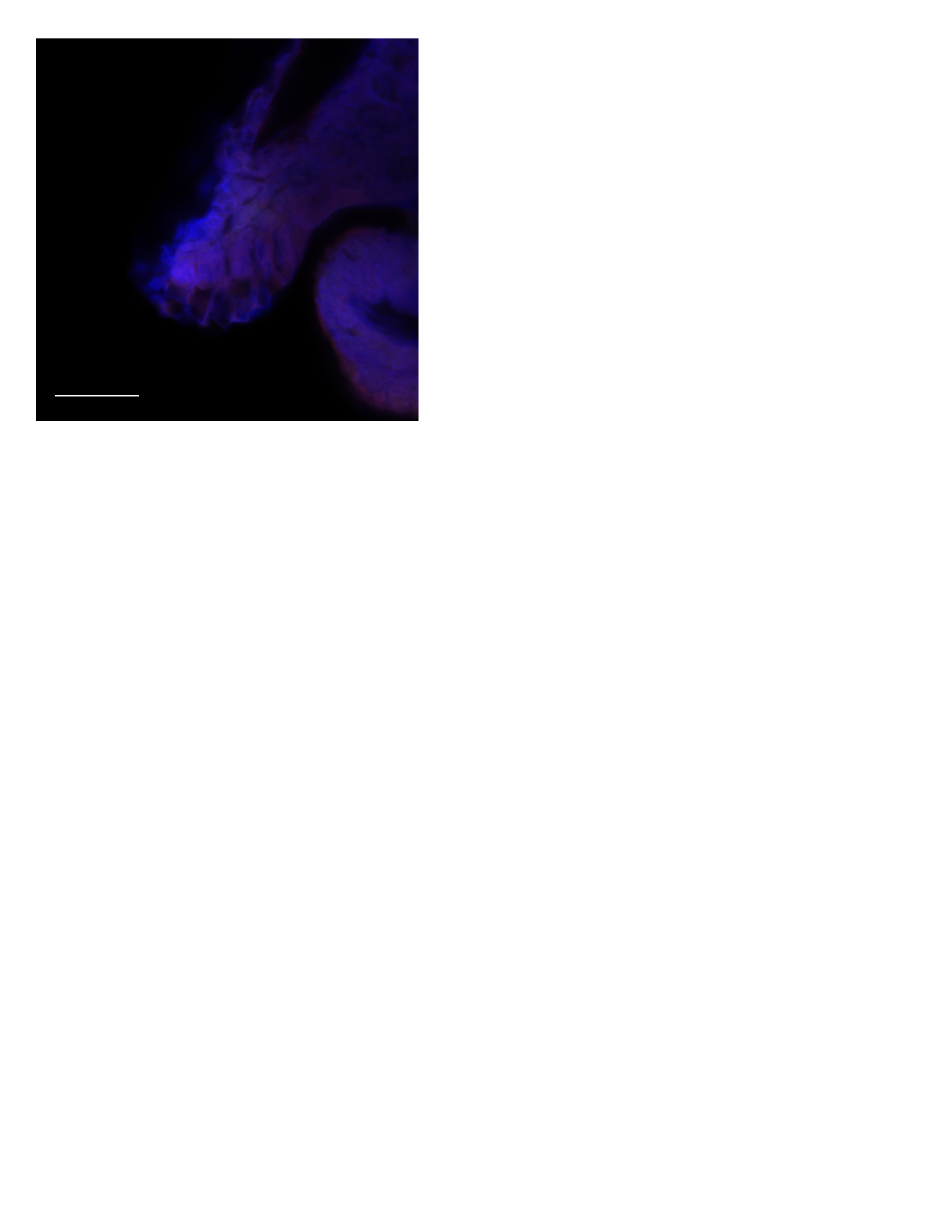

Supplement: S5 Fig — Blue represents autofluorescence excited by a 405nm laser. Scale bar, 25μm. (TIF) [file pone.0135381.s005.tif]

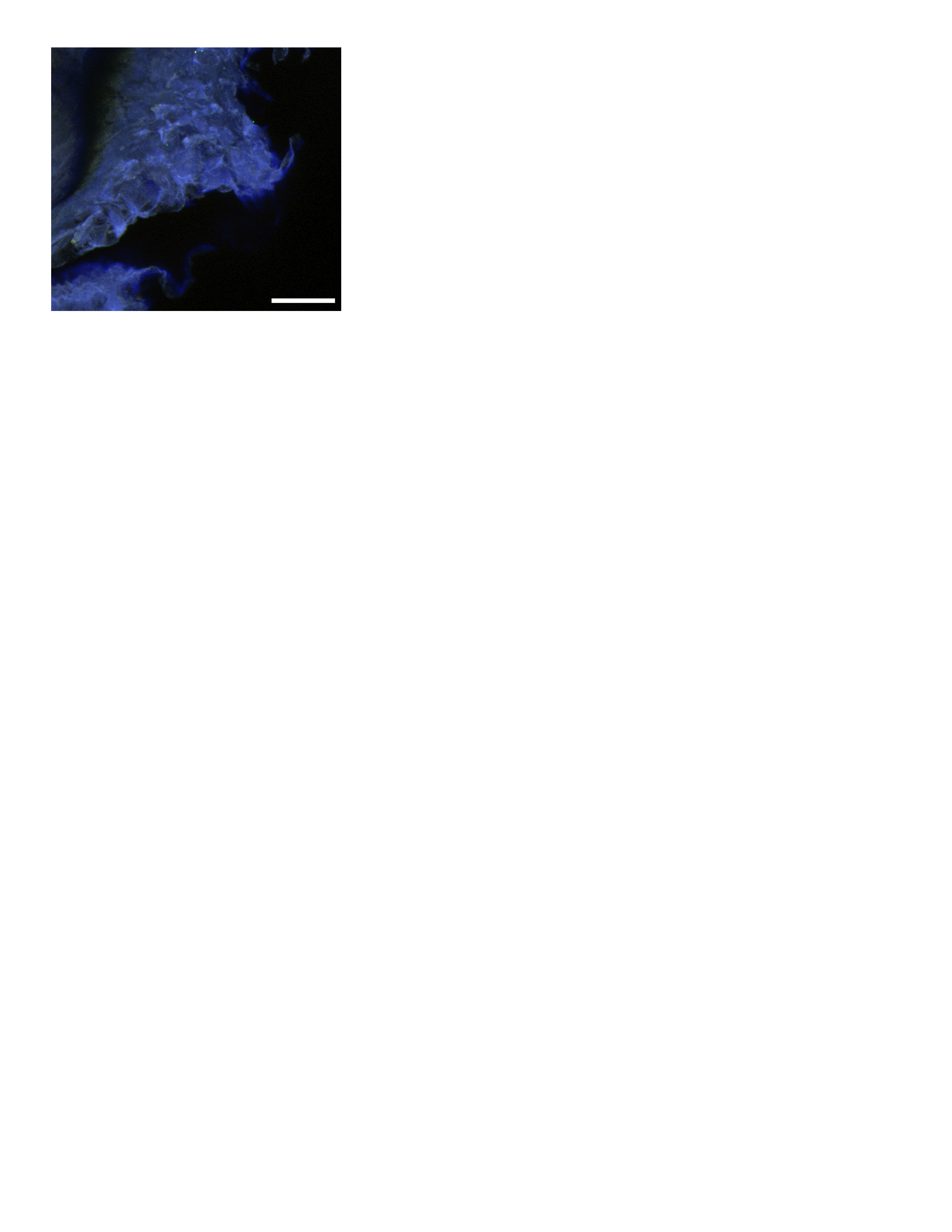

Supplement: S6 Fig — Blue represents autofluorescence excited by a 405nm laser. Scale bar, 25μm. (TIF) [file pone.0135381.s006.tif]
